# Integrated proteomic and phosphoproteomic analysis reveals the MAPK cascade as a key regulator of ethylene-induced latex production in *Hevea brasiliensis*

**DOI:** 10.1007/s44154-026-00290-9

**Published:** 2026-02-19

**Authors:** Linling Yang, Boxuan Yuan, Fengyan Fang, Minmin He, Wei Li, Shugang Hui, Xiaoyu Du, Lixia He, Huijiao Lui, Tian Sang, Xuchu Wang

**Affiliations:** 1 Key Laboratory of Plant Resources Conservation and Germplasm Innovation in Mountainous Region, Ministry of Education, Institute of Agro-Bioengineering, College of Life Sciences, Guizhou University, Guiyang, 550025 China; 2 Institute of Advanced Biotechnology, Institute of Homeostatic Medicine, and School of Medicine, Southern University of Science and Technology, Shenzhen, 518055 China; 3 College of Agriculture Forestry Ecology, Shaoyang University, Shaoyang, 422000 China

**Keywords:** Proteomic, Phosphoproteomic, Latex, Ethylene, Rubber tree

## Abstract

Natural rubber is a crucial industrial raw material globally, with wide applications in industries such as automotive and healthcare. Application of ethylene promotes latex production in the Hevea rubber tree (*Hevea brasiliensis*). However, its potential protein-regulation mechanism remains unclear. To elucidate the role of ethylene in latex production, we employed high-resolution proteomics and phosphoproteomics to determine the global changes in protein abundance and phosphorylation during ethylene-stimulated latex production. Using latex samples collected from ethephon-treated rubber tree, we identified more than 3,700 quantifiable proteins and 2,000 phosphorylated proteins with over 6,000 phosphorylation sites. Proteins involved in the mitogen-activated protein kinase (MAPK) cascade, metabolic pathways, and vesicular trafficking, like rubber elongation factors (REFs), small rubber particle proteins (SRPPs), and 14–3-3 proteins, exhibit pronounced robust phosphorylation dynamics upon ethephon application. Interestingly, the serine/threonine–proline (S/T-P) motif, which could be recognized by MAPKs, was highly enriched in REFs, 14–3-3, and ubiquitin-associated DENN (UDENN) domain-containing proteins, suggesting a potential role of MAPKs in ethylene-induced rubber biosynthesis. Consistently, the MAPKs show altered activation in ethephon-treated Hevea rubber trees, compared to water-treated controls. Additionally, activation of UDENN domain proteins implicated Ras-related Rab GTPases in membrane trafficking and rubber particle formation. These results provide comprehensive information on global changes in protein abundance and phosphorylation upon ethephon application in latex production, and may provide valuable clues for understanding the molecular basis of ethephon-regulated natural rubber biosynthesis in Hevea rubber trees.

**Supplementary Information:**

The online version contains supplementary material available at 10.1007/s44154-026-00290-9.

## Introduction

Natural rubber (NR), a vital biopolymer composed of *cis*−1,4-polyisoprene chains, is primarily synthesized in the laticifers of *Hevea brasiliensis* primarily via the mevalonate (MVA) pathway (Amerik et al. 2018; Cao et al. 2025; Cherian et al. 2019). This process involves the condensation of isopentenyl pyrophosphate (IPP) monomers catalyzed by rubber transferase complexes anchored on rubber particles, along with auxiliary interacting proteins, ultimately forming polyisoprene chains that account for over 90% of latex dry weight (Cherian et al. 2019; Liu et al. 2020; Tang et al. 2016; Men Xiao et al. 2018; Wadeesirisak et al. 2023; Southorn and Edwin 1968). Latex regeneration occurs continuously within laticifers and is initiated by tapping, which is a mechanical wounding procedure that triggers latex release (Fan et al. 2022; Buakong et al. 2023). However, the limited yield improvement from traditional tapping techniques has led to the widespread use of ethylene stimulation. (e.g., ethephon application) in rubber plantations (Zhu et al. 2009; Pellegrin et al. 2004). Since the 1960 s, ethylene treatment has been shown to increase latex yield by 1.5–twofold, through the dual mechanisms of prolonging latex flow and promoting laticifer regeneration (Coupé and Chrestin 1989; Audley et al. 1976; Liu 2016).

Proteomic and transcriptomic studies have partially deciphered ethylene’s role in latex metabolism. Ethylene accelerates glycolysis to supply precursors for rubber biosynthesis while suppressing chitinase-mediated latex coagulation (Amalou et al. 1992; Yang et al. 2024a). Concurrently, it enhances sucrose metabolism and upregulates aquaporins to optimize osmotic balance and water transport in laticifers, thereby extending latex flow (Liu 2016; Wu et al. 2019; Liu et al. 2023; Wang et al. 2013; Zou et al. 2024). Notably, ethylene stimulation induces significant metabolic alterations in latex without eliciting pronounced transcriptional changes, suggesting that post-translational modifications (PTMs) may act as critical regulatory switches in ethylene signaling—a hypothesis that remains underexplored (Yang et al. 2024a).

Phosphorylation, a reversible PTM, plays a pivotal role in regulating enzyme activity, protein–protein interactions, and cellular signaling pathways (Day et al. 2016; Gong et al. 2022; Zhang et al. 2023). In plants, phosphorylation networks are extensively involved in hormone signaling, metabolic adaptation, and environmental stress responses (Li et al. 2015; Sang et al. 2025). Ethylene signaling induces the activation of mitogen-activated protein kinase 3 and 6 (MPK3/6), which catalyzes the phosphorylation of the transcription factor SCREAM (SCRM), a functional homolog of inducer of CBF expression 1 (ICE1). This modification enhances SCRM's transcriptional activity, promoting its binding to cis-regulatory elements in the *WUSCHEL-related homeobox 8* (*WOX8*) promoter. This mechanism drives asymmetric division of the Arabidopsis zygote and initiates embryonic polarity establishment (Chen et al. 2025). Similarly, liquid chromatography-tandem mass spectrometry (LC–MS/MS)-based quantitative phosphoproteomics has revealed that ethylene-induced phosphorylation of membrane proteins under high humidity conditions modulates stomatal movement and reactive oxygen species (ROS) homeostasis via the ethylene insensitive 2 (EIN2)/ethylene insensitive 3 (EIN3) signaling module. In this context, phosphorylation of ethylene biosynthesis genes (1-aminocyclopropane-1-carboxylate synthase (ACS), 1-aminocyclopropane-1-carboxylate oxidase (ACO)) serves as an early event in humidity sensing (Jiang et al. 2024). Furthermore, in cotton, combined proteomic and phosphoproteomic analyses demonstrated that the scaffold protein ghirsutum mitogen-activated protein kinase organizer 1 (GhMORG1) confers resistance to *Fusarium oxysporum* by enhancing the activity of the gossypium hirsutum mitogen-activated protein kinase kinase 6 (GhMKK6)-ghirsutum mitogen-activated protein kinase 4 (GhMPK4) cascade and facilitating phosphorylation of thirty-two downstream substrate proteins (Wang et al. 2020).

In rubber trees, pioneering studies identified 59 ethylene-responsive phosphoproteins associated with calcium signaling and redox homeostasis (Wang et al. 2015). However, the use of low-throughput techniques such as two-dimensional gel electrophoresis (2-DE) and difference in-gel electrophoresis (DIGE) limited mechanistic resolution. Recent advances in LC–MS/MS combined with data-independent acquisition (DIA) have revolutionized phosphoproteomics (Benschop et al. 2007; Sugiyam et al. 2008). DIA employs fixed-window cyclic fragmentation of all precursor ions, eliminating the stochastic ion selection bias inherent to data-dependent acquisition (DDA), and greatly enhancing cross-batch reproducibility (Lin et al. 2018). This approach achieves high coverage while standardizing fragmentation patterns to minimize technical variability, ensuring high quantitative consistency across samples (Kitata et al. 2023). Although DIA-based phosphoproteomics has successfully identified early signaling events in model plants (e.g., Arabidopsis, Tomato) under cold and osmotic stress, its application to latex biology remains unexplored (Sang et al. 2024; Tan et al. 2025). Furthermore, conventional phosphoproteomic approaches often conflate phosphorylation-specific dynamics with changes in protein abundance, a limitation that can be overcome by integrating global proteomics with phosphoproteomic profiling (Yang et al. 2024b; Wang et al. 2022; Zander et al. 2020).

In this study, we employed DIA-based quantitative phosphoproteomics combined with global proteomics to elucidate the role of ethephon in enhancing latex yield. This strategy overcomes prior methodological constraints by distinguishing phosphorylation-specific dynamics from protein abundance variations. Our results demonstrate that ethephon treatment strongly activates the MAPK signaling pathway during latex biosynthesis. Furthermore, key phosphoproteins involved in rubber biosynthesis, such as small rubber particle protein (SRPP), rubber elongation factor (REF), 14–3-3 proteins, and critical enzymes in the MVA pathway, displayed pronounced phosphorylation changes. Notably, MAPK-recognized [S/T-P] motifs were identified on REF, 14–3-3 proteins, and UDENN domain-containing regulators, providing the first mechanistic evidence directly linking ethylene-activated MAPK signaling to rubber production. These findings not only deepen our understanding of metabolic signaling in rubber tree latex but also establish a molecular framework for enhancing rubber yield and quality through molecular breeding and genetic engineering. Collectively, this work delineates novel mechanisms of ethylene-regulated latex metabolism, offering new perspectives for studying phytohormone-controlled metabolic networks.

## Results

### Proteomic and phosphoproteomic insights into ethephon-induced latex synthesis in *Hevea brasiliensis*

In our previous studies, we observed that ethephon treatment significantly increased latex yield in *H. brasiliensis* (rubber tree) and accelerated the formation of small rubber particle organelles. Due to the technological limitations at the time, we employed a proteomics approach using two-dimensional electrophoresis (2-DE) combined with in-gel digestion to preliminarily identify the latex proteome and phosphorylation modifications under ethephon response (Wang et al. 2015). However, the number of identified proteins and phosphorylation sites was relatively limited due to the resolution and sensitivity of traditional techniques, resulting in insufficient coverage of low-abundance regulatory proteins. Here, we utilized data-independent acquisition (DIA) technology combined with a high-resolution mass spectrometry platform to conduct in-depth proteomic and phosphoproteomic analyses of ethephon-treated latex samples. By integrating multi-omics data, this study will provide new molecular targets for deciphering the high-yield mechanism of rubber trees and offer theoretical support for the sustainable production of natural rubber.

**Fig. 1:**
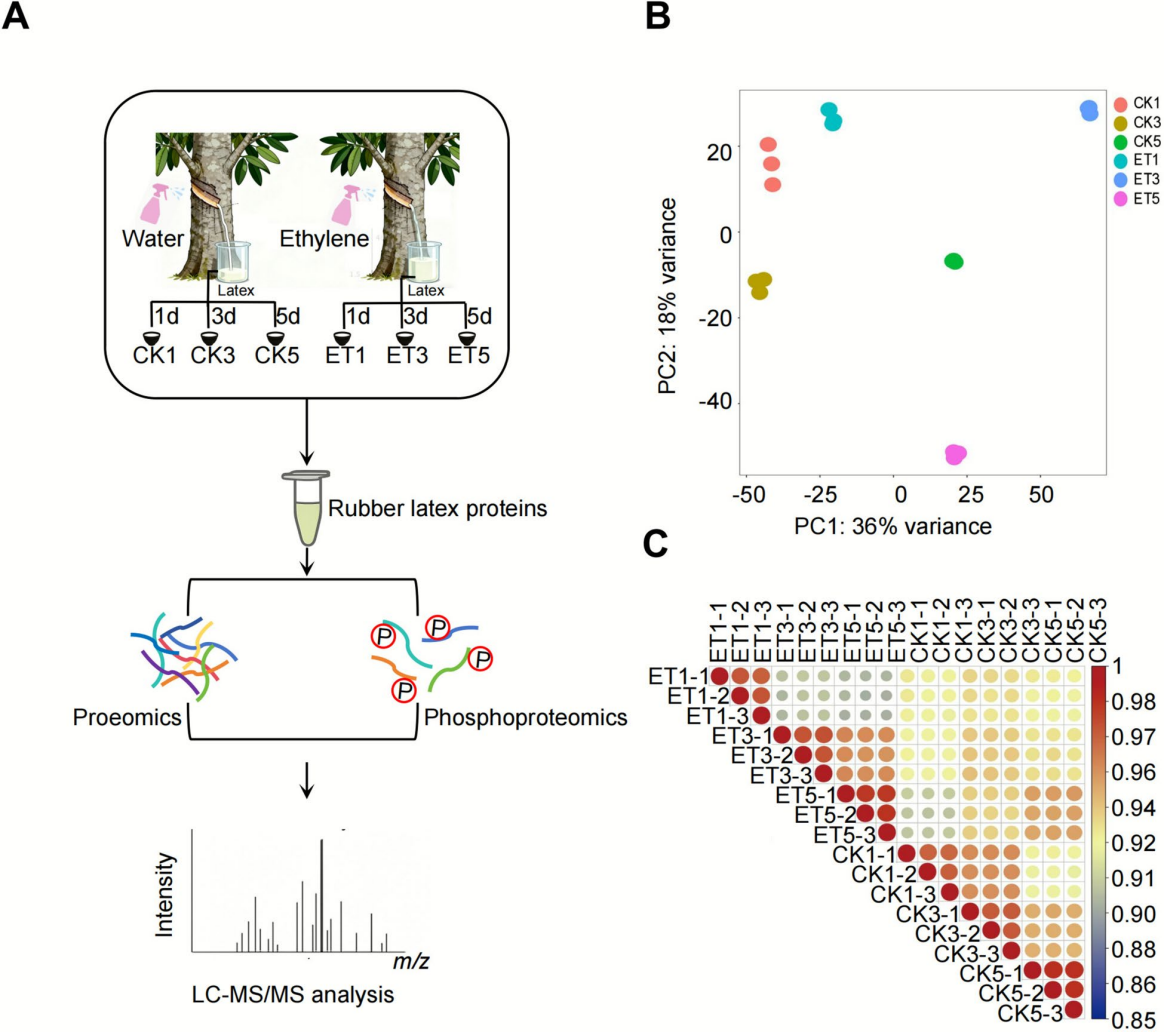
Proteomic and phosphoproteomic analysis of latex from ethephon-stimulated rubber trees. **A**. experimental workflow for proteomic and phosphoproteomic profiling. Latex samples were collected from rubber trees (ten-year-old *H. Brasiliensis* Mull. Arg., clone RY 7–33–97) treated with 3% ethephon (ET) or ultrapure water (control, CK) at 1-, 3-, and 5-days post-treatment (designated as ET1, ET3, ET5 and CK1, CK3, CK5, respectively). Total proteins were extracted, digested, and analyzed by LC–MS/MS for proteomic profiling. A subset of peptides was further enriched for phosphopeptides to obtain phosphoproteomic data. **B**. principal component analysis (PCA) of proteomic data. The plot displays the distribution of biological replicates across different treatment groups (CK1, CK3, CK5, ET1, ET3, ET5), illustrating intra-group reproducibility and inter-group variations in protein expression induced by ethephon stimulation. **C**. pearson correlation analysis of the whole proteome of rubber tree latex under ethephon treatment

We selected healthy and consistent untreated rubber trees (clone RY 7–33–97) and treated the tapping surfaces with 3% ethephon (ET, experimental group) or ultrapure water (UPW, control group). Latex samples were collected on days 1, 3, and 5 after treatment for protein extraction and comparative proteomics and phosphoproteomics analyses. Using high-resolution DIA-based proteomics and phosphoproteomics, we identified changes in protein abundance and phosphorylation status in latex (Fig. 1A). Principal component analysis (PCA) revealed clear separation between ET-treated and control samples, indicating substantial treatment-induced proteomic shifts (Fig. 1B). Across all samples, 3,700 proteins were quantitatively identified (Supplementary Table S1). In the correlation analysis, control groups showed consistent profiles, whereas latex proteins exhibited significant differential expression between three and five days post-ethephon treatment (Fig. 1C).

### Ethephon-induced proteomic changes in rubber tree

**Fig. 2:**
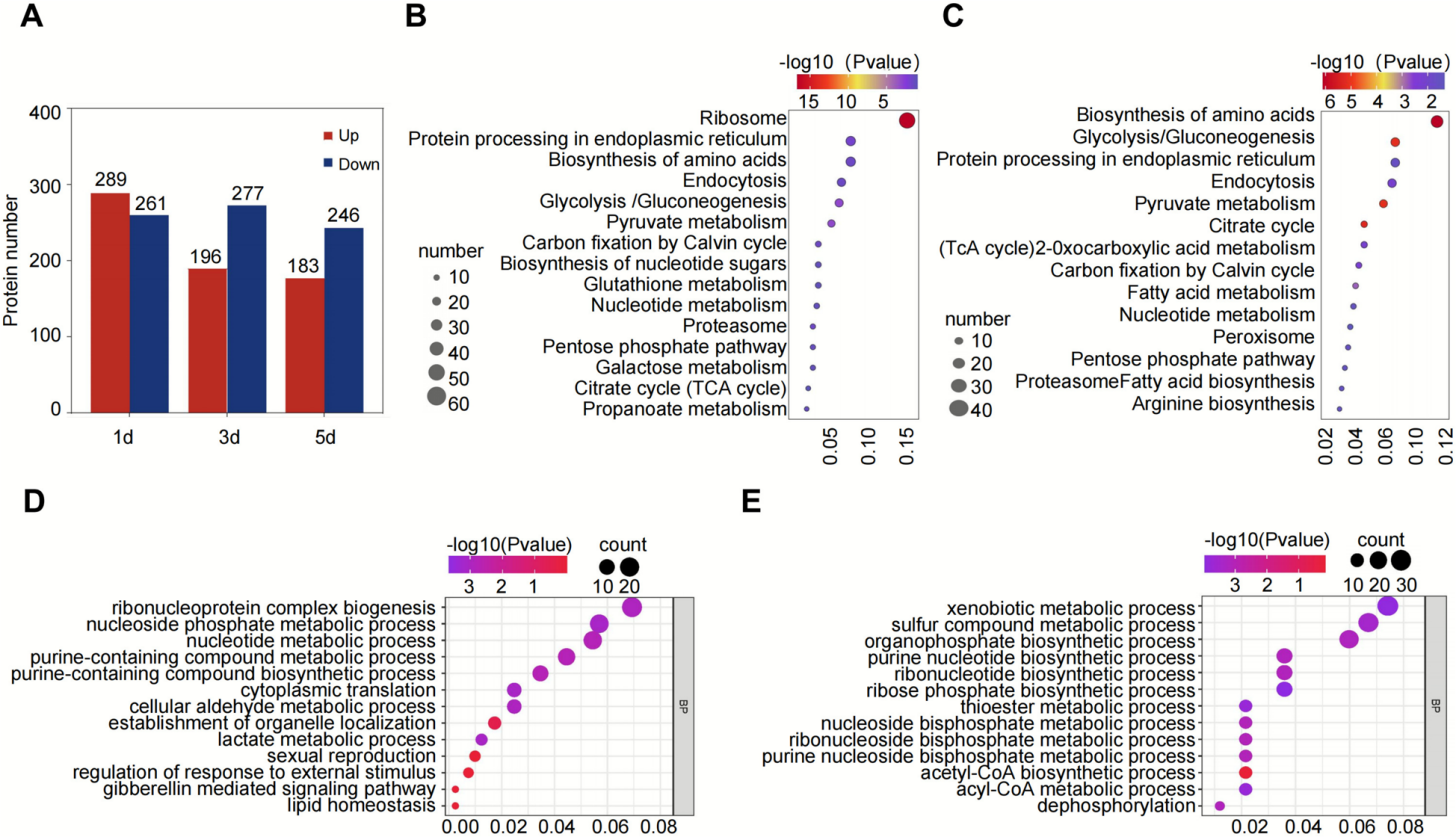
Differential protein expression and functional analysis in response to ethephon treatment in rubber tree latex. **A**. the number of differentially expressed proteins. Red represents upregulated proteins (log2 (FC) ≥ 0.585, *P* < 0.05), and blue represents downregulated proteins (log2 (FC) ≤ −0.585, *P* < 0.05). **B**. KEGG pathway enrichment analysis performed on the combined dataset of proteins upregulated at 1, 3, and 5 days after ethephon treatment. The top 15 enriched pathways are shown, with the y-axis representing the -log10 (*p*-value) for each pathway. Pathways such as ribosome biogenesis, protein processing in the endoplasmic reticulum, and biosynthesis of amino acids were significantly enriched. **C**. KEGG pathway enrichment analysis performed on the combined dataset of proteins downregulated at 1, 3, and 5 days after ethephon treatment. The top 15 enriched pathways are displayed, with the y-axis representing the -log10 (*p*-value) for each pathway. Key enriched pathways include biosynthesis of amino acids, glycolysis/gluconeogenesis, and protein processing in the endoplasmic reticulum. **D**. significantly enriched biological processes (GO-BP terms) for proteins upregulated in response to ethephon treatment at 1, 3, and 5 days. The top 13 enriched BP pathways based on statistical significance are shown. **E**. significantly enriched biological processes (GO-BP terms) for proteins downregulated in response to ethephon treatment at 1, 3, and 5 days. The top 13 enriched BP pathways based on statistical significance are shown

Proteomic analysis of *H. brasiliensis* latex revealed extensive changes in protein expression in response to ethephon treatment. A total of 1,452 differentially expressed proteins (DEPs) were identified, indicating significant changes following ethephon treatment. Of these, 289, 196, and 183 proteins were upregulated on days 1, 3, and 5 after treatment, respectively, while 261, 277, and 246 proteins were downregulated at the corresponding time points (Fig. 2A, Supplementary Table S2).

To explore the biological processes underlying the ethephon-induced increase in latex yield, Gene Ontology (GO) (Supplementary Fig. S2A, Supplementary Table S3) and Kyoto Encyclopedia of Genes and Genomes (KEGG) pathway enrichment analyses were performed on the DEPs (Fig. 2B, C, Supplementary Table S4). Among the upregulated proteins, the most enriched categories were those involved in nucleic acid metabolism, response to external stimuli, signal transduction regulation, and translation-related biological processes (Fig. 2D, Supplementary Table S3). Molecular functions such as metal ion binding, antioxidant activity, and protein degradation regulation were significantly enriched. In cellular components, the endoplasmic reticulum and proteasome-related complexes were notably enriched (Supplementary Fig. S2A). For the downregulated proteins, enrichment was observed in metabolic pathways, particularly xenobiotic metabolism, sulfur compound metabolism, and nucleic acid metabolism (Fig. 2E, Supplementary Table S3). Similarly, molecular functions such as metal ion binding, antioxidant activity, and protein degradation were also significantly enriched. Cellular component analysis revealed that the differential proteins were primarily localized in the proteasome, endoplasmic reticulum, and plastid outer membrane (Supplementary Fig. S2B). KEGG pathway analysis showed that after ethephon treatment, upregulated proteins were mainly concentrated in protein processing, amino acid metabolism, carbohydrate metabolism, and nucleotide metabolism pathways (Fig. 2B). In contrast, downregulated proteins were enriched in fundamental metabolic pathways such as carbohydrate metabolism, fatty acid metabolism, and photosynthesis (Fig. 2C).

### Phosphoproteomic analysis of ethephon-induced modifications in *H. brasiliensis* latex proteins

Although the proteomic analysis provided insights into the functional distribution of proteins involved in ethephon-induced latex production, interpreting the dynamic regulatory network of this process remains challenging when relying solely on static protein abundance data. To elucidate the activity of latex proteins under ethephon stimulation and the signaling pathways involved, we employed the DIA phosphoproteomics approach to investigate the phospho-mediated signaling triggered by ethephon in rubber tree latex. Latex proteins from trees treated with ethephon or water for 1, 3, and 5 days were enzymatically digested, and phosphopeptides were enriched using immobilized metal affinity chromatography (IMAC). For this purpose, we digested latex proteins from rubber tree latex treated with ethephon or water for 1, 3, and 5 days, followed by phosphopeptide enrichment using the IMAC method. The enriched phosphopeptides were subsequently analyzed and quantified by DIA mass spectrometry.

**Fig. 3:**
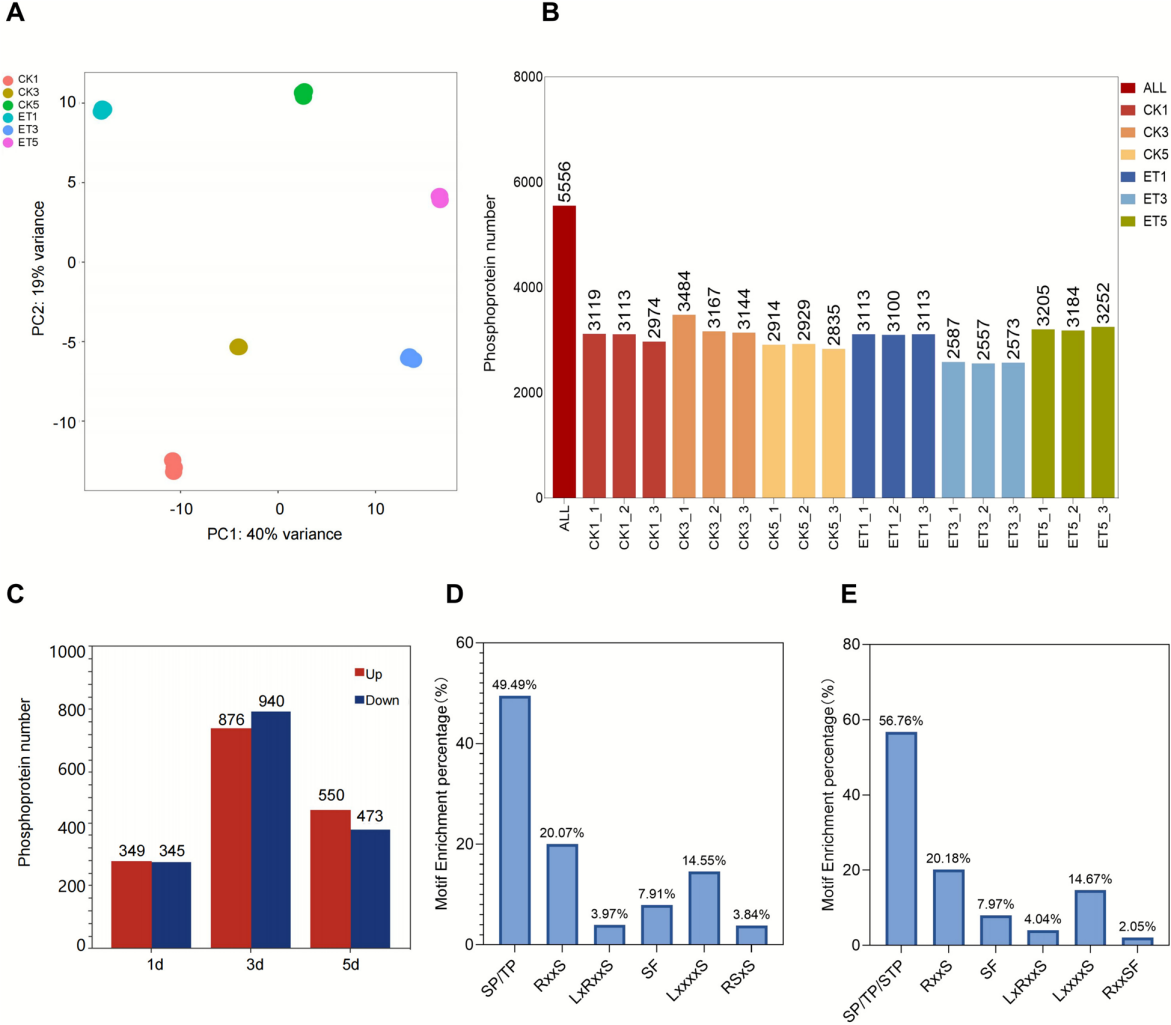
Phosphoproteomic analysis of rubber tree latex under ethephon treatment. **A**. principal component analysis (PCA) of the phosphoproteomic data, showing distinct clustering of the experimental groups (ET1, ET3, ET5) and the control groups (CK1, CK3, CK5) based on the phosphoproteome. This analysis highlights the variability between treatments and demonstrates the significant impact of ethephon treatment on phosphoprotein expression. **B**. bar plot of identified phosphorylated proteins across groups, presenting the number of phosphorylated proteins detected in each treatment group. **C**. the red bars represent upregulated phosphorylated proteins, and the blue bars represent downregulated proteins. This data provides insights into the dynamic changes in phosphorylation across time points. The number of differentially phosphorylated proteins identified at different time points (days 1, 3, and 5) in response to ethephon treatment. Red represents up-enriched phosphoproteins (log2 (FC) ≥ 1, *P* < 0.01), and blue represents down-enriched phosphoproteins (log2 (FC) ≤ −1, *P* < 0.01). **D**. statistical analysis of the enriched motifs in upregulated phosphopeptides. The percentage of different motifs found in the upregulated phosphopeptides is presented, with the most frequent motifs identified, showing the specific sequence patterns that are enriched following ethephon treatment. **E**. statistical analysis of the enriched motifs in downregulated phosphopeptides. This bar graph shows the percentage of motifs enriched in the downregulated phosphopeptides, providing insights into how phosphorylation patterns are altered in response to ethephon treatment

In total, 7,369 unique phosphopeptides were identified, corresponding to 5,550 phosphorylation sites on 2,013 proteins (Supplementary Fig. S1C). For quantitative analysis, we retained only proteins detected in at least 70% of the samples, ensuring stability and representativeness of the latex phosphoproteome. We assessed the sample quality and reproducibility of each condition by conducting principal component analysis (PCA) on the phosphopeptides identified in 18 replicate experiments. The results indicated that the six conditions were distinct from each other, but the biological replicates of each condition clustered together (Fig. 3A), demonstrating high reproducibility and unique phosphoproteomic profiles for each condition. Phosphorylation analysis of amino acid residues showed that phosphorylated serine (p-Ser), phosphorylated threonine (p-Thr), and phosphorylated tyrosine (p-Tyr) residues were 4,716, 620, and 219, respectively, accounting for 84.90%, 11.16%, and 3.94% of the total phosphorylation sites (Supplementary Fig. S1A), which are the most conserved phosphorylation sites in plants. Additionally, in comparing proteomics and phosphoproteomics, 751 proteins were found to overlap between the proteome and phosphoproteome (Supplementary Fig. S1B).

We observed that ethephon treatment significantly modulated the phosphoproteome of latex. Specifically, 349, 876, and 550 phosphoproteins exhibited increased phosphorylation abundance at 1 day, 3 days, and 5 days post-ethephon treatment (*P* < 0.01, log2 (FC) ≥ 1) (Fig. 3C, Supplementary Table S5), whereas 345, 940, and 473 phosphoproteins demonstrated decreased phosphorylation abundance (*P* < 0.01, log2 (FC) ≤ −1) (Fig. 3C, Supplementary Table S5). Given that phosphorylation sites can serve as indicators of kinase activity (Müller-Dott et al. 2025), we performed motif enrichment analysis on 1,775 upregulated and 1,758 downregulated phosphosites. This reveals a significant enrichment of motifs such as (-R-x-x-pS-x-S), a potential substrate for MEKK, MAP2K, or MAPK. Furthermore, motifs such as (-x-pS-P-x-), (-x-pT-P-x-), and (-x-pS-T-P-) were significantly enriched in both upregulated and downregulated phosphopeptides (Supplementary Fig. S4A, B), together accounting for approximately 50% of all identified motifs (Fig. 3D, E). These motifs were recognized by kinases such as MAPK, cyclin-dependent kinases (CDKs), and glycogen synthase kinase 3 (GSK-3), which regulate diverse processes including cell cycle progression, stress responses, metabolic pathways, and protein degradation.

### Dynamic analysis and clustering of differential phosphoproteins

**Fig. 4:**
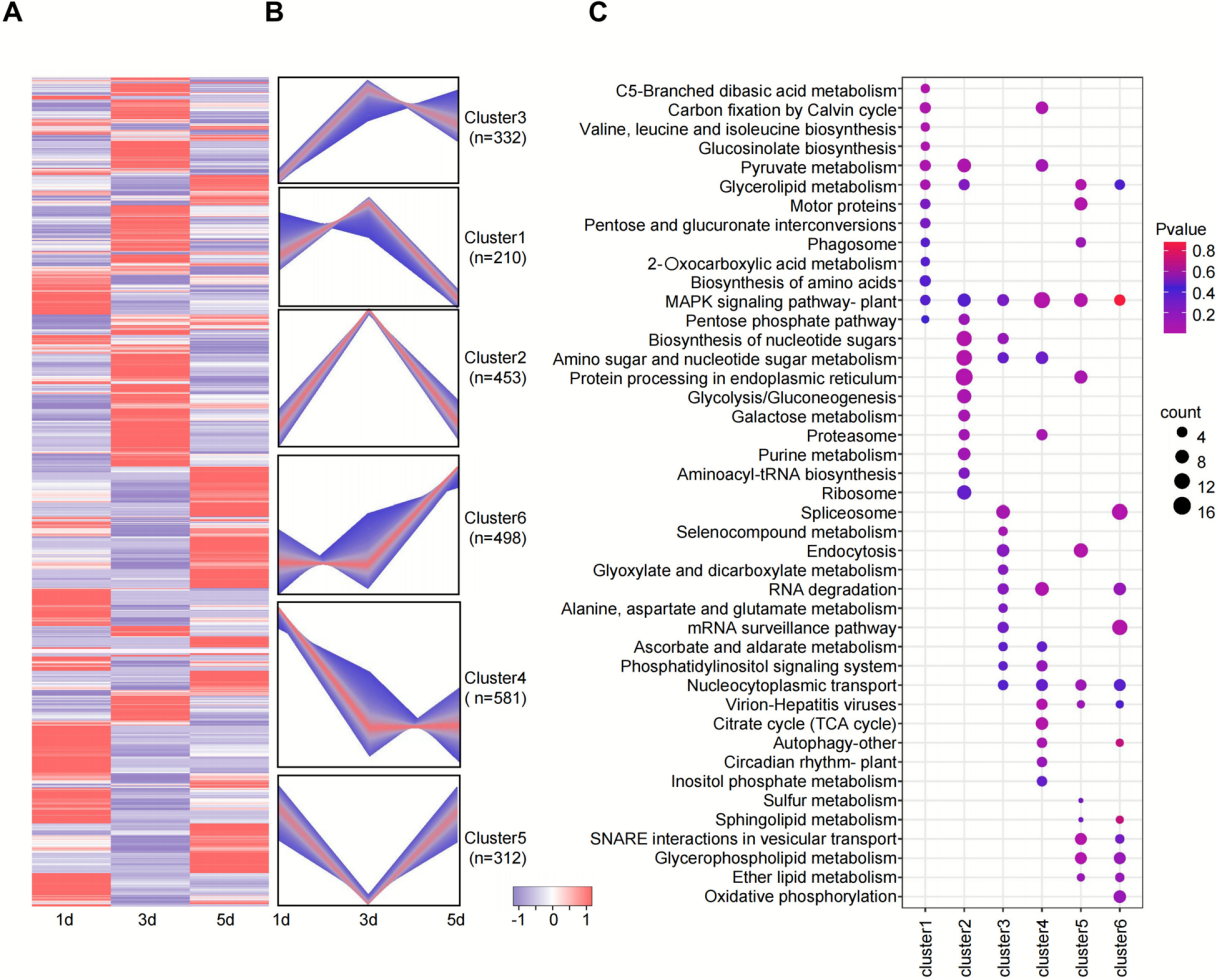
Dynamic clustering analysis of phosphorylated proteins in the phosphoproteome under ethephon treatment. **A**. heatmap showing hierarchical clustering analysis of phosphopeptide Z-score intensities in ethephon-treated samples at 1 d, 3 d, and 5d. **B**. dynamic clustering analysis comparing phosphopeptide responses in ethephon-treated samples at 1 d, 3 d, and 5d. **C**. bubble matrix plot displaying the KEGG enrichment results for each cluster

**Fig. 5:**
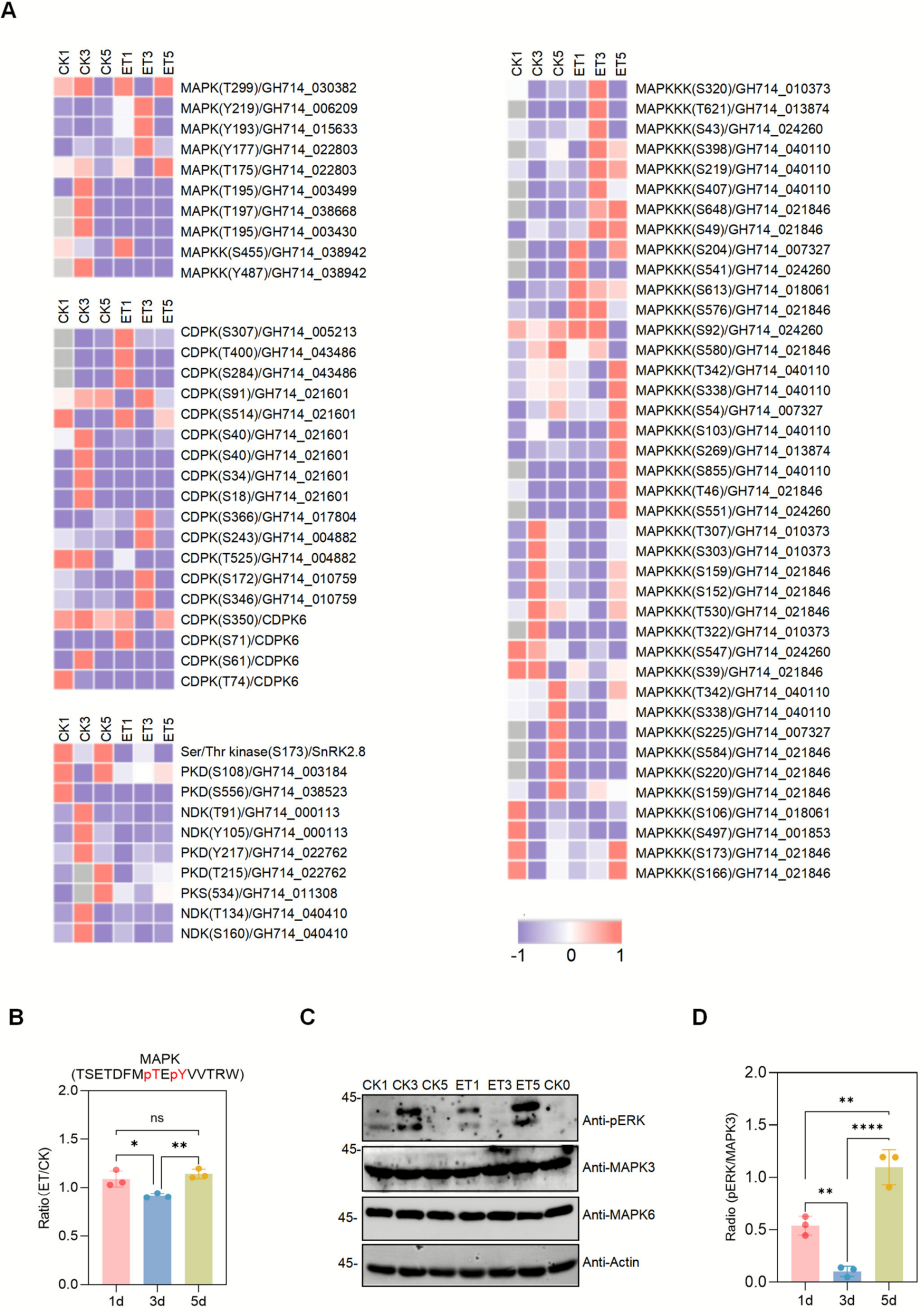
MAPK cascade activation by ethephon. **A**. heatmap showing relative abundance of phosphopeptides within the phosphoproteome of MAPKs, MAPKKKs, MAPKKs, and CDKs under ethephon treatment. The color intensity indicates the z-score intensity. The horizontal axis represents different sample groups, and the vertical axis represents gene names. **B**. the bar graph illustrates temporal changes in MAPK protein phosphorylation levels following ethephon treatment, expressed as the ratio of ethephon-treated to control samples (ET/CK). The x-axis indicates the post-treatment time points (1d, 3 d, 5 d), while the y-axis represents the phosphorylation ratio (ET/CK). Ratios > 1 indicate phosphorylation upregulation, whereas ratios < 1 denote downregulation. Each bar displays the mean value of three biological replicates (*n* = 3), with error bars representing the standard deviation. **C**. immunoblot analysis showing the phosphorylation of MAPKs upon ethephon treatment. Protein extracts were probed with antibodies against pERK1/2, pMPK3, and pMPK6. Anti-actin immunoblotting was used as a loading control. The images shown represent data from three independent experiments. MAPK, mitogen-activated protein kinase; MAPKKK, MAPK kinase kinase. **D**. the pERK1/2 western blot signals were normalized to total MPK3 protein levels to correct for variations in loading. The graph shows the relative phosphorylation levels after normalization (mean ± SD, *n* = 3). MPK3 was chosen as the internal loading control due to its stable expression across the experimental conditions tested. Statistical analysis was performed using one-way ANOVA with Tukey’s multiple comparison post-test: ns (not significant, *P* ≥ 0.05), * P < 0.05, ** P < 0.01, *** P < 0.001, **** P < 0.0001. Data represent mean ± SEM (*n* = 3 biological replicates)

To further elucidate the role of ethephon stimulation in latex synthesis signaling, we performed hierarchical clustering and GO enrichment analysis on upregulated and downregulated phosphorylation sites and proteins across 1, 3, and 5 days after treatment. Z-score normalization grouped the phosphoproteins into six distinct clusters (Cluster 1-Cluster 6) (Fig. 4A, Supplementary Table S6). Functional enrichment analysis of proteins in each of the six clusters revealed significant enrichment of the MAPK signaling pathway in all clusters (Fig. 4B and C, Supplementary Table S7). Specifically, in Cluster 1 and Cluster 3, proteins related to the MAPK signaling pathway showed elevated phosphorylation levels at 1 and 3 days, accompanied by enrichment of pathways related to cell cycle and metabolic regulation. In contrast, in Cluster 5 and Cluster 6, phosphorylation of MAPK pathway proteins peaked at 5 days, suggesting a potential role in sustaining long-term regulation of latex synthesis. Heatmap analysis of the phosphorylation levels of MAPK pathway-related proteins further supported the time-dependent dynamic changes of these proteins during ethephon treatment (Fig. 5A, Supplementary Table S8). Taken together, the significant enrichment of the MAPK signaling pathway across all clusters and its temporal changes across different clusters indicate that this pathway plays an important role in latex synthesis under ethephon stimulation. Under phosphorylation modification, the MAPK signaling pathway may regulate the expression of proteins involved in latex synthesis at early stages, and later promote long-term latex production by regulating processes such as metabolism and the cell cycle.

To validate our proteomics findings, we performed western blotting with antibodies that recognize the phosphorylated T-x–Y motif, a hallmark of MAPK activation. As shown in the Fig. 5, phosphorylation of MPK3 and MPK6 was induced after ethephon treatment (Fig. 5B, C, D, Supplementary Table S9). It is noteworthy that we observed a transient decrease in the phosphorylation level of MAPK kinases at the day 3 (ET3), which may represent a feedback inhibition mechanism elicited by rubber trees in response to ethephon stimulation. For instance, potent and sustained hormonal stimuli can induce the expression of specific phosphatases to modulate the amplitude and duration of the signaling (Farooq and Zhou 2004; Owens and Keyse 2007). Nonetheless, Integration of the proteomics and western blot data confirmed that ethephon activates multiple components of the MAPK cascade. In addition, phosphoproteomic analysis revealed that ethephon stimulation also triggered activation of several calcium-dependent protein kinases (CDPKs) (Fig. 5A; Supplementary Table S8), as evidenced by increased phosphorylation at multiple serine/threonine sites.

These findings strongly support that the MAPK cascade plays a crucial regulatory role in the ethylene response, primarily through phosphorylation-dependent activation. Given the well-established involvement of the MAPK pathway in stress responses, cell proliferation, and developmental processes, we propose that phosphorylation-mediated activation of MAPK signaling contributes to the ethylene-induced modulation of metabolic activity, energy supply, and, ultimately, enhanced latex production in rubber tree cells.

### Phosphoproteins involved in natural rubber biosynthesis pathway

**Fig. 6:**
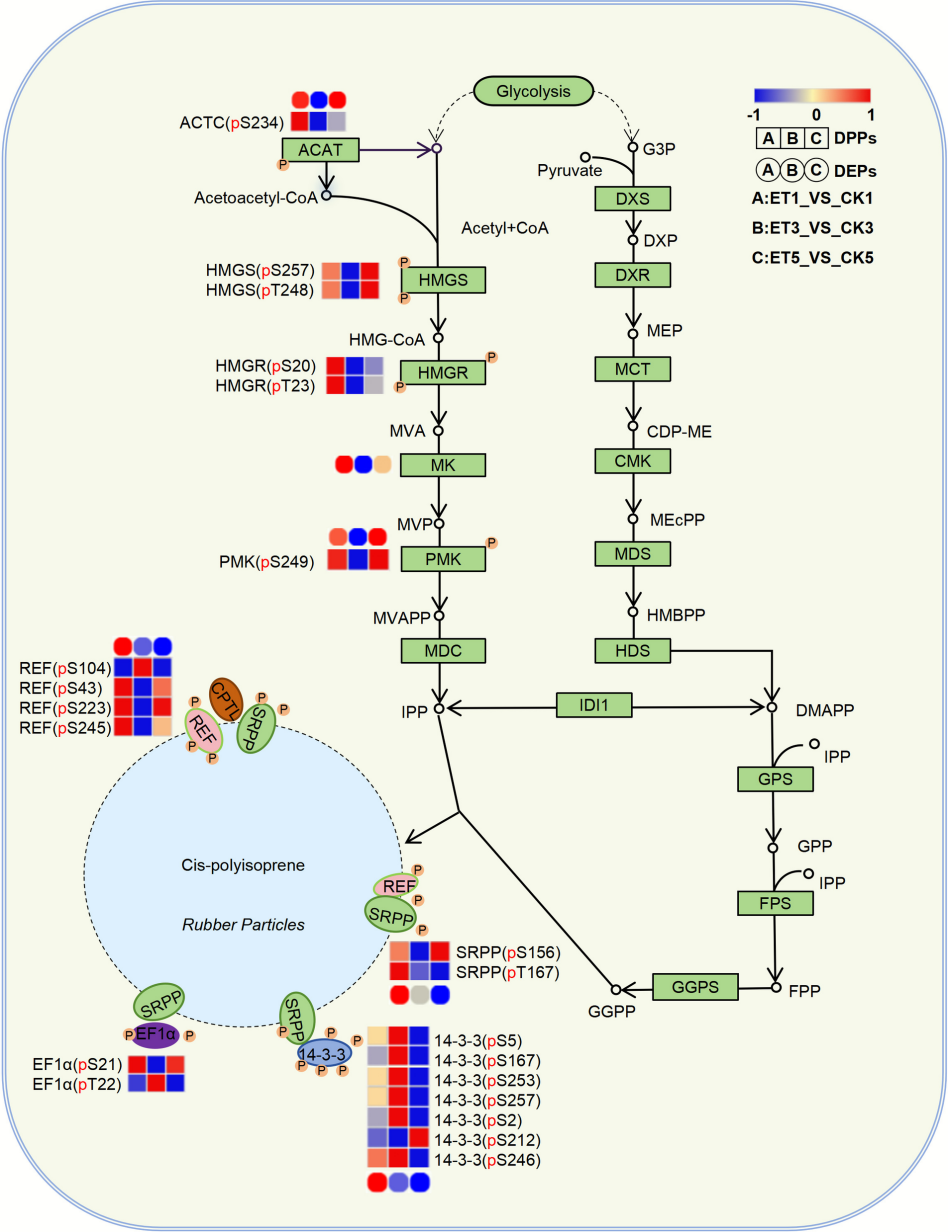
Dynamic modulation of the rubber biosynthesis pathway in *Hevea brasiliensis* latex upon ethephon treatment. The heatmap illustrates temporal changes in protein abundance (circles) and phosphorylation levels (squares) of key proteins involved in the mevalonate (MVA) and methylerythritol phosphate (MEP) pathways, as well as rubber particle-associated proteins, at 1, 3, and 5 days after 3% ethephon application. Color intensity reflects the direction and magnitude of change: red indicates upregulation, blue indicates downregulation, and white denotes no significant change relative to the respective control

The synthesis of natural rubber relies on the production of the precursor IPP, which is subsequently polymerized and extended on rubber particles through the action of proteinases, forming cis-1,4-polyisoprene long chains, the primary component of natural rubber (Cherian et al. 2019; Liu et al. 2020; Tang et al. 2016; Amerik et al. 2018). Ethephon stimulation has been shown to enhance the yield of natural rubber latex (Wu et al. 2019). However, functional enrichment analysis of the phosphoproteins in latex that respond to ethephon stimulation did not reveal any enriched functional terms directly related to IPP synthesis (Supplementary Table S3−4). To further investigate the phosphorylation effects of ethephon stimulation on the rubber synthesis pathway and associated proteins, we analyzed the changes in the phosphorylation levels of proteins involved in the MVA pathway, SRPP, REF, and other rubber biosynthesis-related pathways after ethephon stimulation. Key enzymes in the MVA pathway, such as 3-hydroxy-3-methylglutaryl-CoA reductase (HMGR), phosphomevalonate kinase (PMK), and others, exhibited significant phosphorylation-level changes at 1, 3, and 5 days post-ethephon treatment (Fig. 6, Supplementary Table S10). Notably, PMK, HMGR, and HMGS exhibited phase-specific phosphorylation patterns: initial upregulation on day 1, downregulation on day 3, and rebound upregulation on day 5. This oscillatory phosphorylation likely represents a homeostatic regulatory mechanism that maintains metabolic equilibrium in rubber-producing laticifers. This dynamic phosphorylation process may represent a mechanism for maintaining metabolic balance within the organism.

In addition to the key enzymes in the MVA pathway, rubber synthesis-related proteins (such as SRPP, REF, and 14–3-3) also exhibited significant changes in phosphorylation levels after ethephon treatment (Fig. 6, Supplementary Table S10). Notably, we provide the first identification of phosphoproteins directly linked to rubber synthesis, such as REF and SRPP, expanding upon previous findings by Wang et al. (2015) and offering a more detailed phosphorylation site profile. For instance, phosphorylation at REF Ser43 and SRPP Ser156 was strongly upregulated at days 1 and 5, but downregulated at day 3, whereas REF Ser104 showed a specific increase at day 3. Such site-specific phosphorylation may influence the assembly or stability of rubber particles, thereby directly affecting rubber biosynthesis. Notably, several phosphorylation sites of 14–3-3 proteins (such as Ser2, Ser167, and Ser246) were significantly upregulated at 1 and 3 days. Their phosphorylation may synergistically regulate the formation of rubber particles and the elongation of rubber chains. Previous studies have shown that 14–3-3, as a conserved protein, interacts with SRPP and REF to influence rubber particle formation and rubber chain elongation, while binding to phosphorylated serine or threonine residues and participating in various signaling pathways. The phosphorylation levels of UDENN domain (Marat et al. 2011), which are associated with rubber particle synthesis and rubber tree latex vessel development, also exhibited corresponding dynamic changes under ethephon stimulation. In-depth analysis revealed that ethephon-responsive phosphorylation sites on key regulatory proteins (REF, 14–3-3, HMGR, UDENN) consistently contain the signature MAPK substrate recognition motif ([S/T]-P). This pivotal discovery establishes a direct mechanistic link between ethylene signaling, MAPK activation, and rubber biosynthesis regulation, providing a novel molecular framework for deciphering ethylene-mediated yield enhancement (Supplementary Fig. S6A, B, Supplementary Table S11).

## Discussion

Ethylene serves as a stimulant for latex production in *H. brasiliensis*. In rubber industry practices, application of ethephon (an ethylene-releasing agent) increases latex yield by 1.5- to twofold (Amerik et al. 2018; Duan et al. 2010; Bakshi et al. 2015; Qin et al. 2023). This yield enhancement primarily stems from accelerated latex regeneration and prolonged latex flow duration (Yang et al. 2024a; Lestari et al. 2018; Suzuki et al. 2012; Dai et al. 2016). Recent studies indicate significant ethylene-induced serine phosphorylation of core rubber particle proteins REF and SRPP subfamilies, implicating phosphorylation modifications in ethylene-mediated effects (Wang et al. 2015). However, the molecular mechanisms underlying ethylene-driven yield improvement remain incompletely elucidated, and the functional implications of ethylene-mediated differential phosphorylation require further investigation.

In this study, we conducted integrated phosphoproteomic and proteomic analyses of latex samples collected at 1, 3, and 5 days after treatment with 3% ethephon. This concentration was optimized to effectively stimulate phosphorylation modifications associated with rubber biosynthesis while minimizing oxidative stress in rubber trees (Wang et al. 2015). Our results reveal that ethephon stimulation activates phosphorylation of multiple components within the MAPK signaling pathway. In parallel, key rubber biosynthesis proteins, including REF, SRPP, HMGR, 14–3-3, UDENN domain-containing proteins, exhibit significant marked phosphorylation changes in response to ethephon. Notably, several phosphorylation sites on these proteins contain the canonical [S/T-P] motif, a potential recognition sequence for MAPKs or other proline-directed kinases such as CDKs. Similarly, phosphorylation of HMGR at [S/T-P] motifs suggests potential MAPK-mediated regulation of IPP biosynthesis via the MVA pathway. These coordinated phosphorylation events, coupled with ethylene-induced reprogramming of core metabolic and developmental pathways, offer new mechanistic insights into the regulation of latex regeneration and yield enhancement.

*H. brasiliensis* is the primary source of natural rubber, with its rubber biosynthesis relying on a complex metabolic network. At the core of this network, the MVA pathway produces isopentenyl diphosphate (IPP), the fundamental monomer of rubber, with the activities of its key enzymes directly determining precursor availability (Wu et al. 2015). In this study, we provide the first evidence that ethephon stimulation induces multi-site phosphorylation of multiple MVA pathway enzymes, including acetyl-CoA acetyltransferase (ACAT), HMGS, HMGR, and PMK, with phosphorylation levels exhibiting highly coordinated dynamic changes. Specifically, phosphorylation of ACAT, HMGR, HMGS, and PMK was significantly upregulated (activated) on day 1 post-treatment, broadly downregulated by day 3, and largely restored to basal levels by day 5 (Fig. 6, Supplementary Table S10). This synchronous phosphorylation dynamics across multiple enzymes likely reflects a global metabolic reprogramming strategy in ethephon-stimulated rubber trees. We propose that such regulation dynamically optimizes the activity and catalytic efficiency of these enzymes, thereby redirecting carbon flux toward IPP biosynthesis. This mechanistic insight explains how ethylene induces the formation of abundant IPP precursors to support accelerated latex regeneration (Liu et al. 2016).

As the rate-limiting enzyme of the MVA pathway, HMGR plays a decisive role in controlling IPP precursor flux (Schaller et al. 1995; Harker et al. 2003). In rubber trees, laticifer-specific *HbHMGR1* expression is ethylene-induced and positively correlates with latex regeneration efficiency (Wu et al. 2018; Chao et al. 2015). Consistently, studies of the Arabidopsis *hmgr1-1* mutant reveal that HMGR deficiency triggers global phosphoproteome reprogramming, affecting proteins involved in water channel activity (e.g., PIP2 family), nitrate/ammonium transport (e.g., NRT3, AMT1-1), and photomorphogenesis (e.g., PHOT2) (Heintz et al. 2012). These changes indicate HMGR’s role in coordinating cellular adaptation to MVA flux fluctuations by remodeling ion homeostasis and energy supply mechanisms. Crucially, our observation of ethylene-induced phosphorylation on MVA pathway enzymes (including HMGR) in *Hevea* suggests a dual regulatory function in that HMGR acts not only as a phospho-regulated target responding to upstream signals but also as a central metabolic switch triggering downstream adaptive reorganization to safeguard IPP flux.

Natural rubber biosynthesis occurs in specialized organelles called rubber particles, which originate from the endoplasmic reticulum (ER) (Southorn 1961; Xu et al. 2025; Laibach et al. 2018). Ethephon treatment has been shown to increase the abundance of small rubber particles (SRPs) in latex (Wang et al. 2015). Our phosphoproteomic data provide novel molecular insights into this process. Differentially phosphorylated proteins were significantly enriched in pathways related to ER protein processing and multi-organelle structural regulation, suggesting that ethephon enhances ER functional activity to support maturation of rubber particle-associated proteins. UDENN proteins function as guanine nucleotide exchange factor (GEF) for Rab GTPases, regulating intracellular membrane trafficking, cargo protein enrichment, and vesicle targeting through Rab activation (Marat et al. 2011; Chrispeels and Herman 2000; Richter et al. 2009). The involvement of Rab GTPases in rubber particle formation has been proposed, with their expression modulated by ethylene (Qin et al. 2011). Our results revealed significant ethylene-induced phosphorylation of UDENN domain-containing proteins (e.g., upregulation at Ser74) (Supplementary Fig. S6A, Supplementary Table S11). Combining UDENN's functional characteristic as a Rab GTPase guanine nucleotide exchange factor (GEF) and the regulatory role of Rab proteins in rubber particle formation, we believe that the phosphorylation modification of UDENN may have a functional association with the increase in small rubber particle numbers under ethephon treatment.

The core structural proteins of rubber particles, REF and SRPP, are crucial for maintaining particle stability and regulating the rate of isoprene polymerization (Fang et al. 2024, 2025; He et al. 2024; Wang et al. 2019). This study confirmed that ethephon stimulation induces phosphorylation modifications of REF and SRPP, revealing distinct modification patterns between the two. REF localizes to the rubber particle membrane and participates in the formation of high-molecular-weight complexes and the assembly of the rubber biosynthetic enzyme complex (Amerik et al. 2018; Ding et al. 2022; Duan et al. 2006). Research on the rhythmic gene expression in laticifers of rubber trees found that its expression exhibits a coordinated pattern with key rubber biosynthesis enzyme genes (e.g., *HRT2*, *CPT*) and is enriched in vascular tissues (Wang et al. 2018; Yamashita et al. 2016). Furthermore, analysis of *HbREF* gene expression across *Brazilian* rubber tree clones with low to high rubber yields indicates a positive correlation between *HbREF* expression and rubber content (Priya et al. 2007). This collective evidence underscores the central role of REF in latex synthesis and optimizing the functional complex of rubber particles. Our study reveals for the first time that ethephon stimulation induces a significant dynamic upregulation in the phosphorylation level of REF protein (Ser104), which is activated on day 1, peaks on day 3, and returns to the baseline level by day 5 (Supplementary Fig. S6A, Supplementary Table S11). Notably, the observed dynamic trend of REF Ser104 phosphorylation coincides with the concurrent changes in latex yield (from our unpublished preliminary data), strongly suggesting that REF (Ser104) phosphorylation may directly participate in regulating rubber synthesis. Additionally, the flanking sequence of the REF (Ser104) phosphorylation site contains the typical MAPK-recognizable motif [S/T-P], implying it may be a potential target of the MAPK pathway. It is well established that phosphorylation modifications can regulate protein–protein interactions or alter enzyme activity to control particle assembly (Tetlow et al. 2004; Li et al. 2023). For instance, in maize, phosphorylation of SSIIa dynamically regulates starch synthesis by modulating enzyme activity and complex assembly status, providing an optimized mechanism for chain elongation and branching. (Mehrpouyan et al. 2021). By analogy, serine phosphorylation of REF may enhance its interaction with rubber particle membranes and associated protein complexes, potentially through alterations in surface charge or conformational changes.

SRPP and REF belong to the REF/SRPP superfamily, sharing conserved domains, yet potentially exhibiting functional divergence. Nevertheless, SRPP is crucial for rubber particle stability. Its depletion (e.g., via *TbSRPP*-RNAi) leads to particle aggregation and impaired CPT enzyme activity (Hillebrand et al. 2018). This stabilizing mechanism likely arises from the steric hindrance effect of SRPP, which forms nanopolymeric structures coating the particle surface. Phosphorylation may further enhance the stability of these polymeric forms and optimize their hydrophobic interaction with the phospholipid layer (Hillebrand et al. 2018; Nawamawat et al. 2011; Berthelot et al. 2014). Consequently, ethephon-induced SRPP phosphorylation may facilitate structural maintenance of rubber particles under stress conditions and provide a stable environment for rubber chain elongation.

Ethephon treatment also induced phosphorylation of 14–3-3 protein, which interacts with both REF and SRPP. As a key adaptor protein, 14–3-3 modulates the function, subcellular localization, and signal transduction of its binding partners through specific recognition of phosphorylated target proteins (Denison et al. 2011; Chevalier et al. 2009; Camoniet al. 2018). In the context of rubber particles, phosphorylated 14–3-3 potentially acts as a molecular bridge. By simultaneously binding phosphorylated REF and SRPP, it could promote the efficient assembly, enhanced stability, and functional coordination of the core protein complex within the rubber particle. Collectively, these findings advance our understanding of the regulation of rubber particles, specialized organelles in *H. brasiliensis* latex, through protein post-translational modifications.

The MAPK signaling pathway is a core mechanism in plants for regulating growth, development, and responses to biotic and abiotic stresses. Previous studies demonstrated that ethephon treatment significantly upregulates the expression of *HbMKK4* (a key MAPK kinase, MAPKK) in *H. brasiliensis* latex within 4 h (Wu et al. 2015). Furthermore, genome-wide analysis identified 20 *HbMPK*, 13 *HbMPKK*, and 167 *HbMPKKK* genes in rubber tree, with several members exhibiting differential expression in ethylene-treated latex (Huang et al. 2025). These findings suggest that HbMKK4 may participate in the early ethylene response by transmitting ethylene signaling to the MAPK cascade. In this study, we observed phosphorylation modifications in MAPKKK, MAPKK, and MAPK components following ethylene stimulation (Fig. 5A, Supplementary Table S8). Critically, immunoblotting analysis revealed, for the first time, that ethylene treatment significantly increases the phosphorylation of the terminal MAPK cascade kinases, MAPK3 and MAPK6, in latex, providing direct evidence of MAPK cascade activation (Fig. 5B, D, Supplementary Table S9). It is noteworthy that a baseline level of MAPK phosphorylation, particularly of MAPK3/6, was detected in the wounding control group (CK3), as shown in Fig. 5C. This observation confirms that mechanical tapping (wounding) itself constitutes an initial and sufficient trigger for MAPK activation, which aligns with the well-established role of wound signaling in plants. The critical finding, however, is that the phosphorylation intensity in the ethylene-treated groups was markedly stronger and more sustained by day 5. This pattern suggests a synergistic interplay between the two signals. Specifically, wounding provides the obligatory primary stimulus, whereas exogenous ethylene application acts to significantly amplify and prolong the MAPK cascade response. This amplified and sustained signaling is likely crucial for driving the extensive downstream reprogramming of both the phosphoproteome and metabolic pathways observed under ethylene stimulation. Moreover, we identified that key proteins intimately associated with rubber biosynthesis, such as REF, HMGR, 14–3-3, and UDENN. All contain the classical [S/T-P] phosphorylation motif (Supplementary Fig. S6B, Supplementary Table S11). This motif is a canonical target for recognition and action by multiple kinases, including MAPKs and others such as CDKs. These results not only corroborate the activation of the MAPK pathway in the ethylene response but also reveal the potential for phosphorylation of key rubber synthesis proteins by MAPKs. Collectively, this provides important supportive molecular evidence that ethylene may regulate rubber biosynthesis through the MAPK signaling pathway.

Employing DIA-based phosphoproteomics, this study systematically delineated the dynamic phosphoproteome alterations underlying ethephon-induced latex yield enhancement in *H. brasiliensis*. Compared to previous studies relying on static sampling or lower-throughput techniques, this approach significantly increased the depth of coverage and analytical throughput for the phosphoproteome. Our findings not only provide novel evidence elucidating ethylene 's regulation of laticifer metabolism and rubber particle formation but also establish the pivotal role of the MAPK signaling pathway in latex synthesis and particle assembly. This work highlights future research priorities to better understand the function of MAPK signaling in these processes. Moreover, the comprehensive phosphoproteomic dataset generated in this study provides unprecedented molecular-level resolution to interrogate post-translational regulatory networks underlying rubber yield enhancement.

## Conclusion

In summary, our integrated phosphoproteomic and proteomic analyses demonstrate that the enhancement of latex yield induced by ethylene involves the coordinated activation of the MAPK signaling pathway and targeted phosphorylation of key proteins related to rubber biosynthesis. These components show high potential as targets for strategies aimed at improving latex synthesis. Future work will prioritize functional validation of the identified phosphorylation events. Using transgenic overexpression and gene editing mediated by CRISPR/Cas9 to target key phosphoproteins (such as REF and HMGR) and MAPK components, we aim to further elucidate the precise roles that specific phosphorylation sites play in regulating yield. This will ultimately provide guidance for molecular breeding strategies to develop varieties of rubber tree with high yield.

## Materials and methods

### Plant materials and treatment

Total latex protein samples were obtained from sixty newly tapped mature rubber plants (ten-year-old *H. brasiliensis* Mull. Arg., clone RY 7–33–97), which were grown at an experimental farm of the Chinese Academy of Tropical Agricultural Sciences in Tunchang City, Hainan Province, China. The trees were randomly assigned to two groups: an ethephon-treated (experimental group) and an ultrapure water (UPW)-treated (control group), with 30 trees per group. Each group was further divided into three subgroups (*n* = 10 per subgroup) corresponding to sampling at 1-, 3-, and 5-days post-treatment. Latex was harvested using the standard S/2 d3 tapping system (half-spiral cut every three days) as described previously (Wang et al. 2015). Cut surfaces of experimental trees were treated with 3% (v/v) ethephon solution, while control trees received an UPW. Latex samples were collected on days 1, 3, and 5 after treatment. Samples from ethephon-treated trees were designated ET1, ET3, and ET5, while control samples were labeled CK1, CK3, and CK5, respectively. For each subgroup, latex from ten trees was pooled to form one biological replicate. Three independent biological replicates were prepared per treatment per time point. The latex droplets were collected in ice-chilled glass beakers, immediately frozen in liquid nitrogen, and stored at − 80 °C for subsequent analysis.

### Extraction of protein for proteomics

The extraction of the total latex protein for the rubber was performed according to the method of previous work (Wang et al. 2015). The resulting protein was resuspended in 8 M urea with 50 mM ammonium bicarbonate. The protein concentration was measured using the Detergent Compatible Bradford Protein Assay Kit (Beyotime). The urea concentration was adjusted to a final concentration of 1 M using 50 mM ammonium bicarbonate. For reduction and alkylation, the denatured protein sample was treated with 10 mM TCEP and 40 mM CAA and incubated at 56℃ for 10 min with shaking at 1100 rpm. Then, 50 µg trypsin was added (The usage ratio of trypsin to target protein is 1:50) and incubated at 37 °C for 16 h. Finally, the digested peptide was dried using vacuum centrifugation. Activate SDB-XC StageTip with 100 μL methanol by centrifugation (centrifuge at 1,000 g for 2 min). Wash with 100 μL 80% ACN contained 0.1% TFA (centrifuge at 1,000 g for 2 min). Equilibrate with 100 μL 0.1% TFA (centrifuge at 1,000 g for 2 min). Acidify tryptic peptides with 10% TFA to a final concentration of 1% TFA. Centrifuge at 16,000 g for 10 min at 4℃ and transfer supernatant to StageTip. Centrifuge at 500 g for 2 min; discard flow-through. Wash twice with 100 μL 0.1% TFA (centrifuge at 500 g for 2 min; discard flow-through each time). Wash twice with 100 μL 5% ACN containing 0.1% TFA (centrifuge at 500 g for 2 min; discard flow-through). Elute peptides with 100 μL 80% ACN containing 0.1% formic acid (centrifuge at 700 g for 3 min). Dry samples using vacuum centrifugation and store at −80 °C.

### Phosphopeptide enrichment

Phosphopeptides were enriched using a modified Fe-IMAC tip protocol (Tsai et al. 2014). Briefly, an in-house IMAC tip was made by plugging a 20-μm polypropylene frit disk into the tip end and packed with 10 mg of nickel-nitrilotriacetic acid silica resin (Qiagen). The packed IMAC tip was inserted into a 2-mL Eppendorf tube. Ni^2+^ ions were first removed by adding 100 mM EDTA (centrifuge at 200 g for 1 min). Next, the tip was activated with 100 mM FeCl_3_ and equilibrated with 1% (v/v) acetic acid at pH 3.0 before loading the sample. The tryptic peptides were dissolved in 1% (v/v) TFA and 80% (v/v) ACN and loaded onto the IMAC tip. Washing steps were performed using 1% (v/v) TFA, 80% (v/v) ACN (centrifuge at 200 g for 1 min), and 1% (v/v) acetic acid (pH 3.0). Subsequently, the Fe-IMAC tip was inserted into an activated desalting SDB-XC (3 M) StageTip. The bound phosphopeptides were eluted onto the activated desalting SDB-XC StageTip using 200 mM NH_4_H_2_PO_4_ and directly eluted into sample vials for LC–MS/MS analysis. The eluted peptides were dried under vacuum.

### LC–MS/MS acquisition for proteomics

LC–MS/MS analysis samples were analyzed on a nanoElute2 LC system coupled to a timsTOF Pro 2 mass spectrometer (Bruker) via a CaptiveSpray nano-electrospray ion source. Phosphopeptides were dissolved in 6 µL 0.1% formic acid (FA) and injected onto a 25 cm × 75 µm ID Pepsep C18 column (1.5 µm, 120 Å; Bruker). Source settings were: capillary voltage 4,500 V, dry gas 3.0 L/min, dry temperature 180 °C. Mobile phases were water with 0.1% FA (A) and acetonitrile with 0.1% FA (B; LC–MS grade, Thermo Scientific). The 60 min gradient was: 2–22% B over 45 min, 22–37% B over 5 min, 37–80% B over 5 min, then 80% B for 5 min (v/v). Ion mobility was acquired over 1/K0 = 0.6–1.6 Vs cm-2 using 32 × 26 Th windows with a ramp time of 100 ms. The MS scan range was m/z 100–1,700. The ion-mobility dimension was calibrated regularly with the Agilent ESI-LC/MS tuning mix (m/z, 1/K0: 622.0289, 0.9917 Vs cm-2; 922.0097, 1.1984 Vs cm-2; 1221.9906, 1.3934 Vs cm-2). Data were acquired in DIA-PASEF mode with 16 DIA-PASEF scans per TIMS-MS cycle.

### LC–MS/MS acquisition for phosphoproteomics

The lyophilized phosphopeptide fractions were re-suspended in Buffer A (0.1% formic acid), and 20μL aliquots of which were loaded into a nano Viper C18 (Acclaim PepMap 100, 75 μm × 2 cm) trap column. The online Chromatography separation was performed on the Easy nLC 1200 system (Thermo Fisher). The trapping, desalting procedures were carried out with a volume of 20 μL 100% Buffer A (0.1% formic acid). Then, an elution gradient of 5–40% Buffer B (80% acetonitrile, 0.1% formic acid) in 90 min was used on an analytical column (PepMap RSLC, 75 μm × 25 cm, 2 μm 100 Å). DIA (data-independent acquisition) mass spectrum techniques were used to acquire tandem MS data on an Orbitrap Fusion Lumos mass spectrometer (Thermo Fisher) fitted with a Nano Easy-Spray ion source. Data was acquired using an ion spray voltage of 2.2 kV. For a full mass spectrometry survey scan, the target value was 1E6 and the scan ranged from 400 to 1,000 m/z at a resolution of 60,000 and a maximum injection time of 64 ms. Based on the mass peak density of the pre-mixed sample, data acquisition is configured with a variable window. Each cycle consists of 60 segmented windows, with an overlap of 0 Da between windows. The maximum injection time for the secondary mass spectrometry ions is 54 ms. The collision cell energy (for High Energy Collision-Induced Dissociation, HCD) is set at 28 eV.

### LC–MS data analysis

Database searches were conducted using Spectronaut 18 software with the UniProt *H. brasiliensis* sequence file (UniProt ID: UP000467840). The search parameters were configured as follows: MS1 mass tolerance was set to 10 ppm, and MS2 tolerance was 0.05 Da. Trypsin was selected as the digestion enzyme, allowing up to one missed cleavage. Carbamidomethylation of cysteine (C) was set as a fixed modification. At the same time acetylation at the protein N-terminus (Acetyl, Protein N-term), oxidation of methionine (Oxidation, M), and phosphorylation of serine, threonine, and tyrosine residues (Phospho, S/T/Y) were designated as variable modifications (for phosphoproteome). To ensure high-confidence phosphosite assignments, a localization probability threshold of ≥ 0.75 was applied, retaining only sites that met this criterion for downstream analysis.

In data processing, missing values were imputed with half-row minima for proteins quantified in ≥ 1 complete biological replicate group. Variance-stabilizing normalization (implemented within differential expression analysis for proteomics 2 (DEP2)) was applied to minimize technical variance. For functional enrichment, the protein database was annotated via eggNOG-mapper (v2) (Cantalapiedra et al. 2021) using sequence homology, assigning GO terms and KEGG IDs to build a custom annotation resource. Protein and phosphoprotein fold change (FC) was calculated as the ratio of the average quantified abundances from three biological replicates between control and treatment groups. Significant changes were defined as Phosphoprotein abundance: | log2 (FC) |≥ 1 with *P* < 0.01, Protein abundance: | log2 (FC) |≥ 0.585 with *P* < 0.05. Significantly differential proteins were identified based on the differential analysis results. These proteins were subjected to GO and KEGG enrichment analyses using the enricher functionin clusterProfiler v4.8 (implemented within DEP2), with the constructed mapping file as input. Analysis was using the OmicShare tool (https://www.omicshare.com/tools/home/report/koenrich.html). For motif enrichment analysis, MEME Suite 5.5.7 was used to identify conserved sequence motifs among upregulated and downregulated phosphopeptides (Cheng et al. 2019). The data were visualized using GraphPad Prism (version 10, GraphPad Software, San Diego, CA, USA), https://www.bioinformatics.com.cn, an online platform for data analysis and visualization (Tang et al. 2023), and Morpheus (https://software.broadinstitute.org/morpheus).

### Immunoblotting

The latex protein solution was combined with loading buffer (100 mM Tris, pH 6.8, 4% SDS, 25% Glycerol, 0.2% BPB, 50 mM DTT, 1 × cOmplete Protease Inhibitor Cocktail) and heated at 95 °C for 10 min. The proteins were separated by 8% SDS-PAGE and subsequently transferred to a polyvinylidene fluoride (PVDF) membrane (microporous, IPVH00010, Immobilon-P, pore size 0.2 μm). The membrane was incubated overnight at 4 °C with primary antibodies against pERK1/2 (Cell Signaling Technology, 9102S, 1:5000), MPK3 (Sigma-Aldrich, M8318, 1 μg/mL), and MPK6 (Sigma-Aldrich, A7104, 0.2 μg/mL) in Tris-buffered saline with Tween 20 (TBST) and 1% nonfat milk or BSA. Immunoblots were probed with an anti-actin antibody (ABclonal, AC009, 1:10,000) as a loading control. Primary antibodies were detected using either anti-rabbit (Bio-Rad, 1,706,515, 0.05 μg/mL) or anti-mouse (Bio-Rad, 1,706,516, 0.05 μg/mL) secondary antibodies conjugated to horseradish peroxidase and visualized using an enhanced chemiluminescence reagent (Shenger).

## Supplementary Information

Supplementary Material 1: Fig. S1 Comprehensive phosphoproteomic analysis reveals the modification site distribution and dataset characteristics. Fig. S2 GO enrichment of up and downregulated proteins in response to ethephon treatment. Fig. S3 Comparison of phosphoproteins under different treatments.Fig. S4 Phosphorylated protein motif enrichment analysis. Fig. S5 Bubble matrix plot displaying the GO enrichment results for each cluster. Fig. S6 Phosphorylation dynamics of rubber biosynthesis-related proteins in response to ethephon treatment.

Supplementary Material 2: Table S1 The number of identified proteins.

Supplementary Material 3: Table S2 Differentially expressed proteins in the proteome.

Supplementary Material 4: Table S3 Upregulated and downregulated protein GO analysis.

Supplementary Material 5: Table S4 Upregulated and downregulated protein KEGG analysis.

Supplementary Material 6: Table S5 Statistical analysis results of differentially phosphorylated proteins in latex following ethephon stimulation at days 1, 3, and 5.

Supplementary Material 7: Table S6 Dataset for cluster and heatmap analysis of differentially phosphorylated proteins under ethephon treatment.

Supplementary Material 8: Table S7GO and KEGG analysis results for clusters of differentially phosphorylated proteins.

Supplementary Material 9: Table S8 A collection of latex phosphoproteins involved in the MAPK cascade and activated by ethephon.

Supplementary Material 10: Table S9 Statistical analysis of the results from quantitative assessment of latex protein levels at 1, 3, and 5 days post-ethephon treatment.

Supplementary Material 11: Table S10 Phosphorylation and level changes of proteins related to the rubber production pathway.

Supplementary Material 12: Table S11 Phosphorylation level changes of latex proteins.

## Data Availability

All mass spectrometry data are accessible through the jPOST platform (Okuda et al. 2025) under accession numbers JPST003928 (Proteome; ProteomeXchange: PXD066389, https://repository.jpostdb.org/preview/38833011668c97a1dbdabb, Password: 3635) and JPST003957 (Phosphoproteome; ProteomeXchange: PXD066388, URL: https://repository.jpostdb.org/preview/45537860768c97a5b1a43b and Password: 5746). Data are available within the article or its supplementary materials. The data on dynamic changes in latex yield that were generated during prior unpublished studies are available from the corresponding author, Prof. Xuchu Wang, upon reasonable scientific request.

## Abbreviations

ABA: Abscisic Acid
AP2/ERF: AP2/ERF domain-containing proteins
CDK: Cyclin-Dependent Kinases
CDPK: Calcium-Dependent Protein Kinase
CK1: Casein Kinase 1
CK2: Casein Kinase 2
EIN2: Ethylene Insensitive 2
EIN3: Ethylene Insensitive 3
ET: Ethephon
FC: Fold Change
GhMKK6: Ghirsutum Mitogen-Activated Protein Kinase Kinase 6
GhMORG1: Ghirsutum Mitogen-Activated Protein Kinase Organizer 1
GhMPK4: Ghirsutum Mitogen-Activated Protein Kinase 4
GO: Gene Ontology
GSK-3: Glycogen Synthase Kinase 3
HMGR: 3-Hydroxy-3-Methylglutaryl-CoA Reductase
HMGS: 3-Hydroxy-3-Methylglutaryl-CoA Synthase
IMAC: Immobilized Metal Ion Affinity Chromatography
IPP: Isopentenyl Pyrophosphate
KEGG: Kyoto Encyclopedia of Genes and Genomes
MAPK: Mitogen-Activated Protein Kinase
MAPKK: Mitogen-Activated Protein Kinase Kinase
MAPKKK: Mitogen-Activated Protein Kinase Kinase Kinase
MS: Mass Spectrometry
MS/MS: Tandem Mass Spectrometry
NR: Natural Rubber
PIP2: Phosphatidylinositol 4,5-Bisphosphate
PKD: Protein Kinase D
PMK: Phosphomevalonate Kinase
REF: Rubber Elongation Factor
ROS: Reactive Oxygen Species
RP: Rubber Particles
SRPP: Small Rubber Particle Protein
SRPs: Small Rubber Particles
UDENN: Ubiquitin-Associated DENN
UPW: Ultrapure Water
