# Supplementary material for: Integrated proteomic and phosphoproteomic analysis reveals the MAPK cascade as a key regulator of ethylene-induced latex production in Hevea brasiliensis
Source: Stress Biol. 2026 Feb 19;6(1):18. doi: 10.1007/s44154-026-00290-9 (PMC12920992; doi:10.1007/s44154-026-00290-9)
Supplement: Supplementary file 1 — Supplementary Material 1: Fig. S1 Comprehensive phosphoproteomic analysis reveals the modification site distribution and dataset characteristics. Fig. S2 GO enrichment of up and downregulated proteins in response to ethephon treatment. Fig. S3 Comparison of phosphoproteins under different treatments.Fig. S4 Phosphorylated protein motif enrichment analysis. Fig. S5 Bubble matrix plot displaying the GO enrichment results for each cluster. Fig. S6 Phosphorylation dynamics of rubber biosynthesis-related proteins in response to ethephon treatment. [file 44154_2026_290_MOESM1_ESM.pdf]

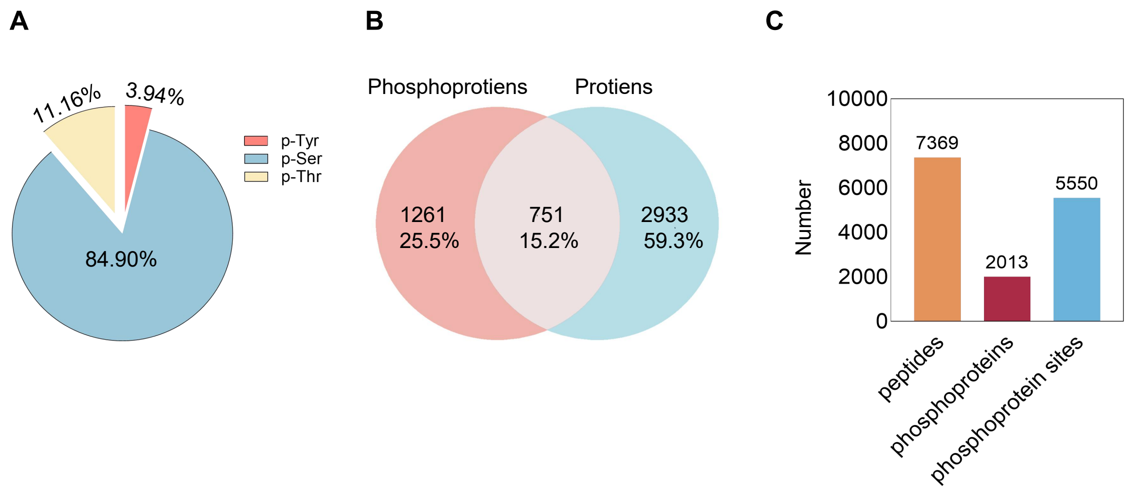

Fig. S1 Distribution of designated phosphorylated amino acid residues for all detected phosphorylation sites. A. pie chart showing the proportion of phosphorylation sites categorized by amino acid type: tyrosine (pTyr), serine (pSer), and threonine (pThr). B. venn diagram displays the intersection of proteins identified in both phosphoproteomic and proteomic analyses. C. bar graph showing the number of identified peptides, phosphoproteins, and phosphoprotein sites in the proteomic and phosphoproteomic analysis

A

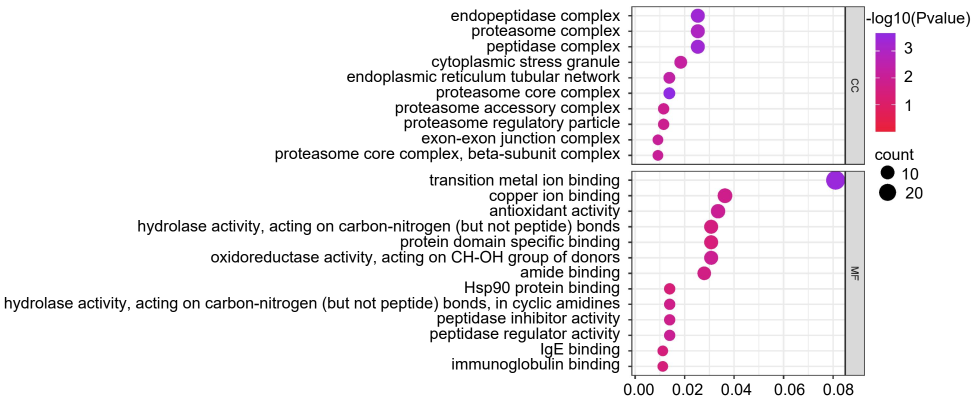

B

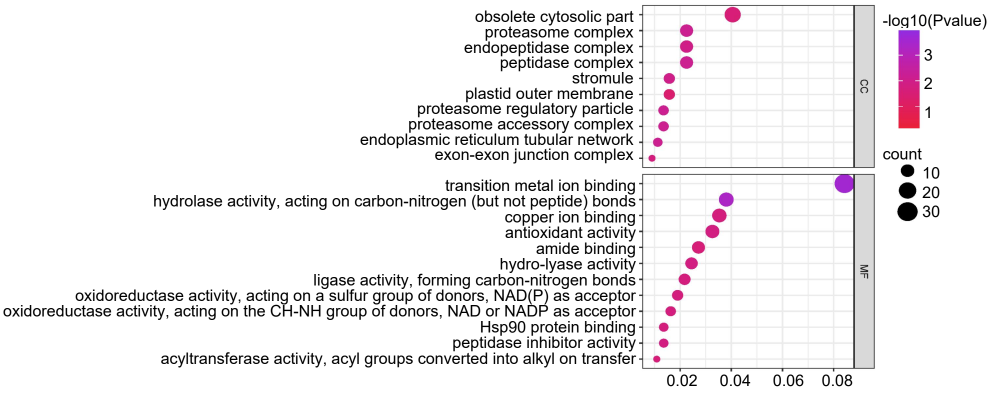

Fig. S2 GO enrichment of up and downregulated proteins in response to ethephon treatment. A. the bubble plot shows the GO enrichment results of upregulated proteins at days 1, 3, and 5 under 3% ethephon treatment (experimental group) compared to ultrapure water treatment (control group). B. the bubble plot shows the GO enrichment results of downregulated proteins at days 1, 3, and 5 under 3% ethephon treatment (experimental group) compared to ultrapure water treatment (control group)

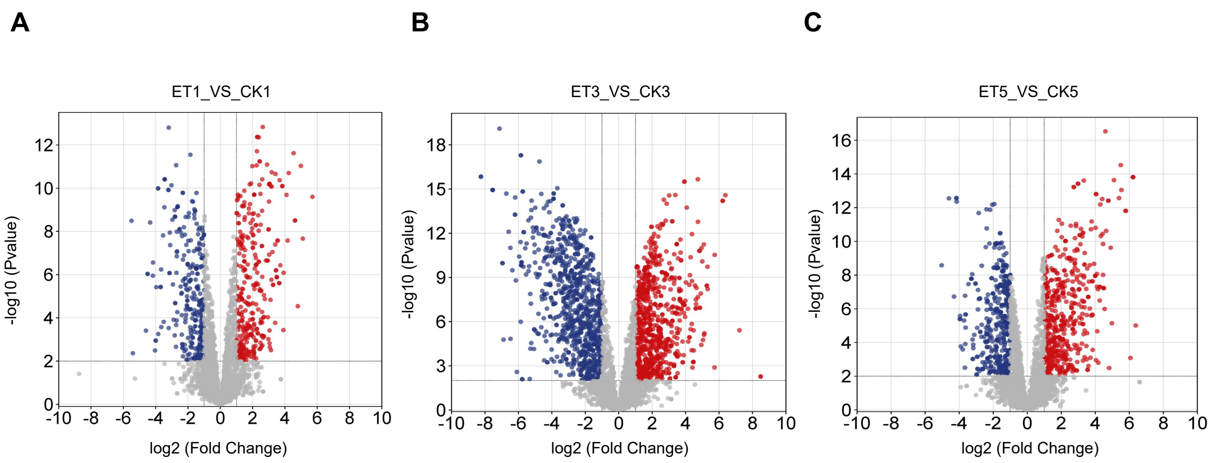

Fig. S3 Comparison of phosphoproteins under different treatments. A- C. volcano plots for differential protein expression in ET1_vs_CK1 (A), ET3_vs_CK3 (B), and ET5_vs_CK5 (C) comparisons. Each volcano plot shows the proteins with significant expression changes under ethephon treatment compared to the control at days 1, 3, and 5, highlighting significantly upregulated and downregulated proteins. The x-axis represents the log2-transformed fold change (FC), and the y-axis represents the negative logarithm of the p-value (-log10 (P-value). Red points indicate significantly upregulated proteins (log2 (FC) ≥ 1, $P < 0.01$), while blue points indicate significantly downregulated proteins (log2 (FC) ≤ -1, $P < 0.01$)

A   B

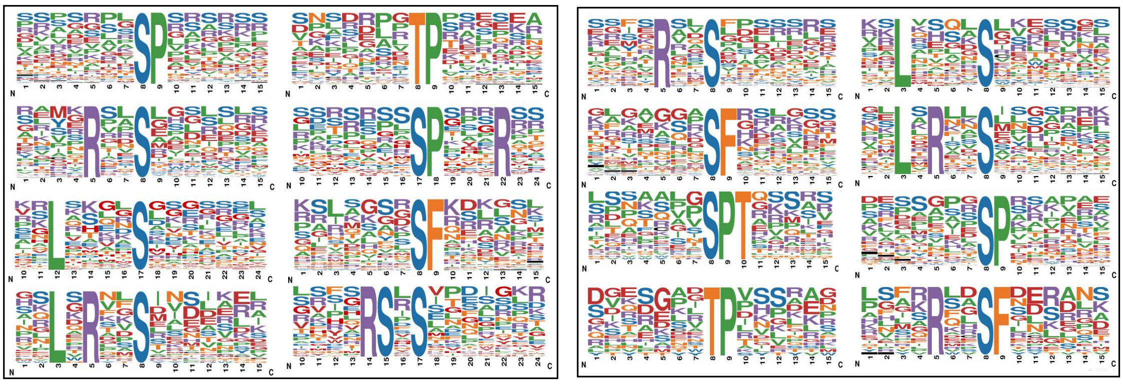

Fig. S4 Phosphorylated protein motif enrichment analysis. A. motif analysis of 1775 upregulated phosphopeptides. B. motif analysis of 1758 downregulated phosphopeptides

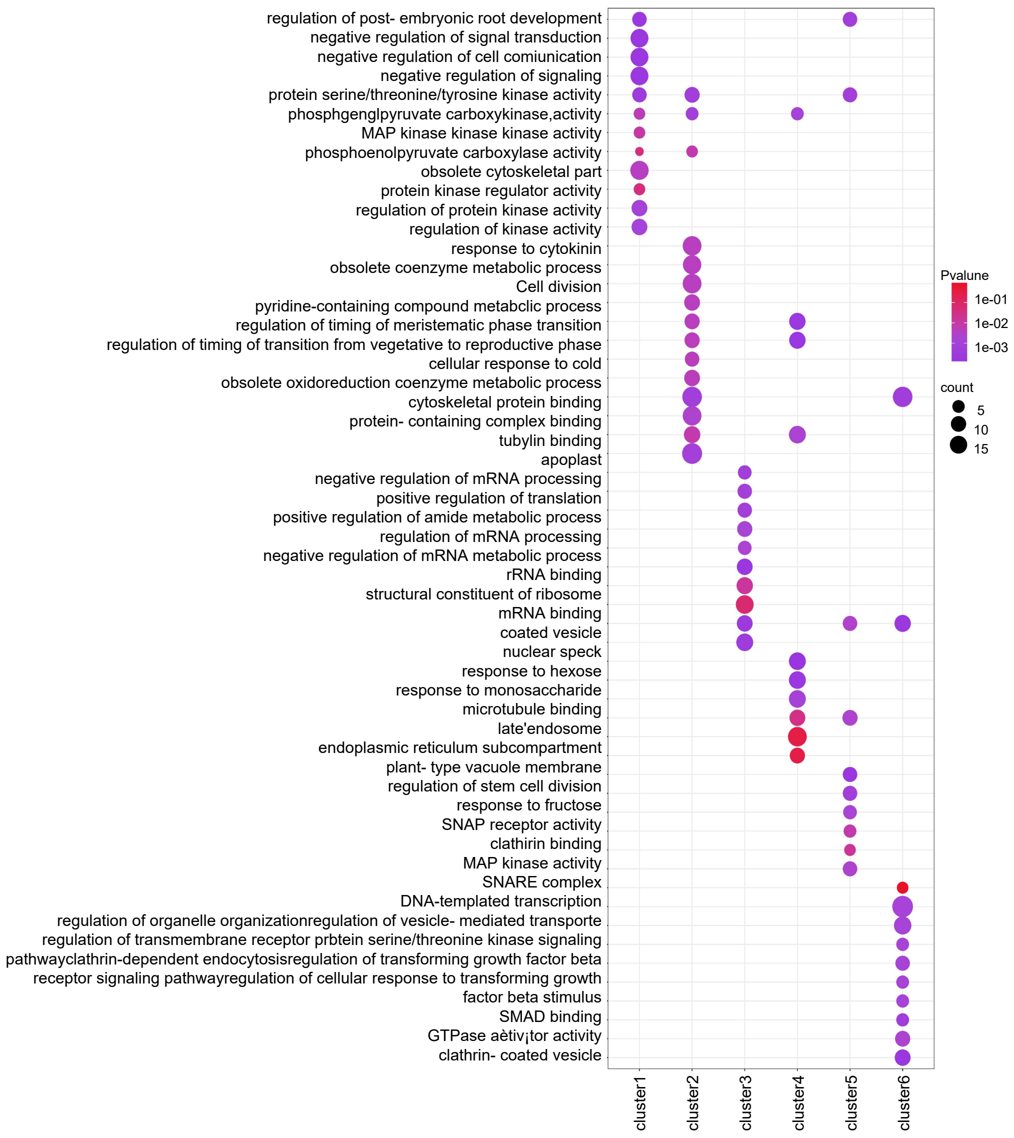

Fig. S5 Bubble matrix plot displaying the GO enrichment results for each cluster. Bubble color indicates the statistical significance of the enrichment, and bubble size corresponds to the number of proteins associated with each GO term. Significantly enriched terms are shown for biological processes, molecular functions, and cellular components

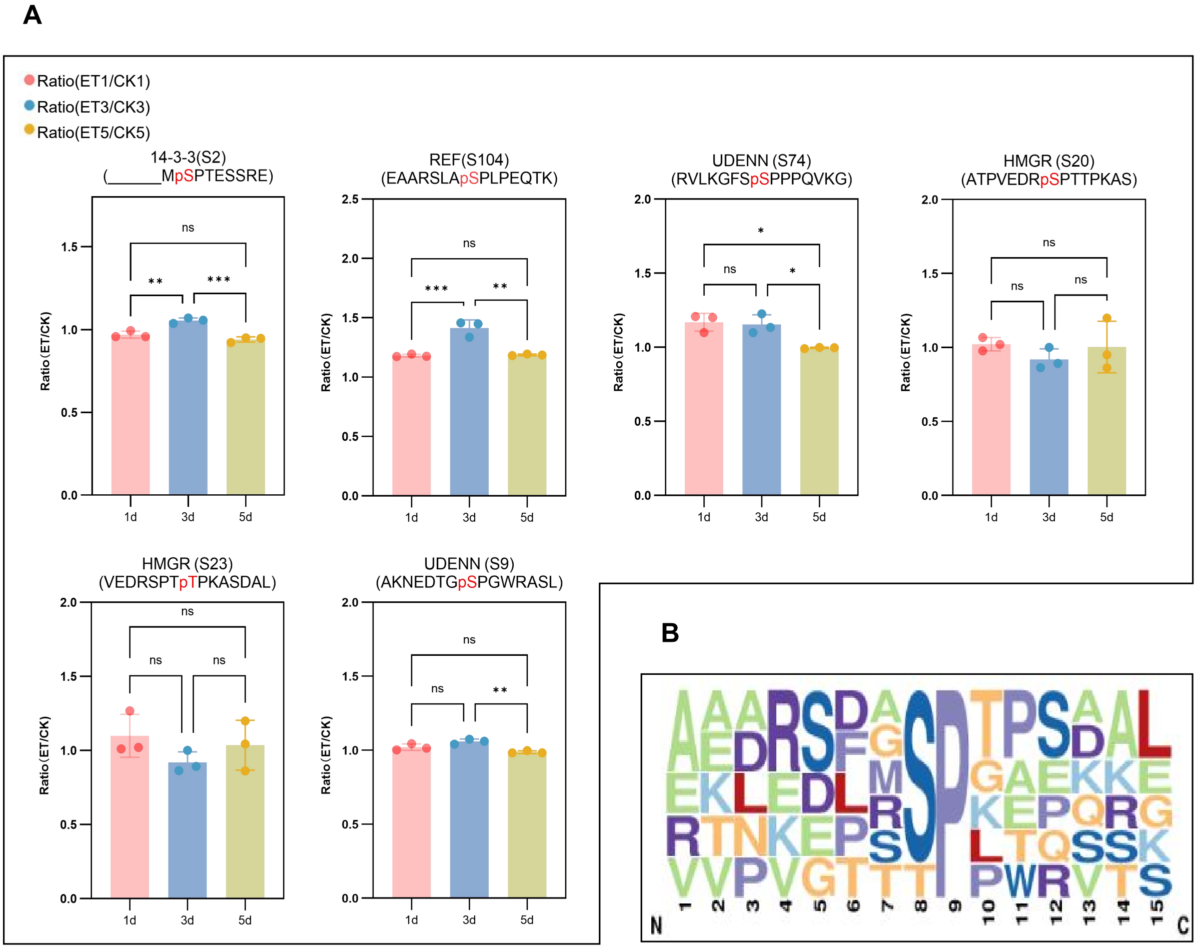

Fig. S6 Phosphorylation dynamics of rubber biosynthesis-related proteins in response to ethephon treatment. A. the figure illustrates temporal changes in phosphorylation levels of key enzymes involved in rubber biosynthesis pathways at 1-, 3-, and 5-days post-ethephon treatment. These proteins possess SP/TP/STP motifs recognized by MAPK kinases. The x-axis indicates the time points (1d, 3d, 5d), while the y-axis represents the phosphorylation ratio between ethephon-treated and control groups (ET/CK). Ratios > 1.0 indicate increased phosphorylation levels, whereas ratios < 1.0 denote decreased phosphorylation. Individual data points (open circles) represent biological replicates (n = 3), with column heights displaying mean ratio values. Statistical analysis was performed using one-way ANOVA with Tukey's multiple comparison post-test: ns (not significant, $P \geq 0.05$), *$P < 0.05$, **$P < 0.01$, ***$P < 0.001$, ****$P < 0.0001$. Data represent mean ± SEM (n = 3 biological replicates). B. motif analysis of 14-3-3, REF, UDENN, and HMGR Proteins
